# Development of a local empirical model of ionospheric total electron content (TEC) and its application for studying solar-ionospheric effects

**DOI:** 10.1038/s41598-021-93496-y

**Published:** 2021-07-23

**Authors:** Pantea Davoudifar, Keihanak Rowshan Tabari, Amir Abbas Eslami Shafigh, Ali Ajabshirizadeh, Zahra Bagheri, Fakhredin Akbarian Tork Abad, Milad Shayan

**Affiliations:** 1 Research Institute for Astronomy and Astrophysics of Maragha (RIAAM), University of Maragheh, Maragheh, Iran; 2 Iranian Space Agency (ISA), Tehran, Iran; 3 Physics Department, University of Tabriz, Tabriz, Iran; 4 Institute for Research in Fundamental Sciences (IPM), School of Particles and Accelerators, Tehran, Iran

**Keywords:** Applied physics, Space physics, Astronomy and astrophysics, Space physics, Space physics, Astronomy and planetary science, Physics

## Abstract

Regular and irregular variations in total electron content (TEC) are one of the most significant observables in ionospheric studies. During the solar cycle 24, the variability of ionosphere is studied using global positioning system derived TEC at a mid-latitude station, Tehran (35.70N, 51.33E). Based on solar radio flux and seasonal and local time-dependent features of TEC values, a semi-empirical model is developed to represent its monthly/hourly mean values. Observed values of TEC and the results of our semi-empirical model then are compared with estimated values of a standard plasmasphere–ionosphere model. The outcome of this model is an expected mean TEC value considering the monthly/hourly regular effects of solar origin. Thus, it is possible to use it for monitoring irregular effects induced by solar events. As a result, the connection of TEC variations with solar activities are studied for the case of coronal mass ejections accompanying extreme solar flares. TEC response to solar flares of class X is well reproduced by this model. Our resulting values show that the most powerful flares (i.e. class X) induce a variation of more than 20 percent in daily TEC extent.

## Introduction

In the Earth’s ionosphere, the variability of space weather is easily reflected in TEC. As the total number of electrons is measured along a vertical column of one square meter cross-section (1 TEC Unit (TECU) $= 1\times 10^{16} \; \; {\rm electrons} \; \; {\rm m^{-2}}$) from the height of a GPS satellite ($\sim 20,000 \;\; {\rm km}$) to the receiver, thus TEC characterizes variations in both ionosphere and plasmasphere^1^.

Until now, various techniques have been used to empirically measure TEC. Some examples include: ionosondes^2,3^, incoherent backscatter radars^2,4,5^, Faraday Rotation (FR) in beacon satellite signals^2,6–8^, altimeter satellite systems, and Global Navigation Satellite Systems (GNSS)^1,9^. The Global Positioning System (GPS) satellites, provide an effective and low-cost method to measure TEC values^10,11^ as a function of time for a specific location on the Earth. GPS signal, propagating through the ionosphere, is advanced in phase and delayed in time. As a result, values of carrier phase and pseudo-range combined L-band frequencies (L1 carrier:1575.42 MHz and L2 carrier: 12227.60 MHz) are used to evaluate TEC^12–15^.

TEC values are subject to both temporal/spatial and regular/irregular variations^16,17^. Spatial variations describe those related to the location on the Earth (i.e. equatorial anomalies etc), whilst temporal variations are related to time (i.e. universal or local). Where, regular variations include periodic changes in TEC values, irregular ones show the temporal effects of phenomena such as solar events and geomagnetic storms.

Investigating TEC variations reveals the main physical processes which are responsible for the ionospheric behaviour. Generally speaking, the changes in TEC values are mainly connected with: the condition of the Earth’s magnetic field, the Earth’s rotation (which induces diurnal effects), the Earth’s position around the Sun (which induces observed seasonal effects) and the solar activity levels. Whilst diurnal and seasonal effects are considered as regular effects, the solar activity levels and its effect on the Earth’s magnetic field may produce both regular and irregular variations. Irregular variations in TEC are mainly due to Traveling Ionospheric Disturbances (TID)^18–20^ and/or ionospheric or geomagnetic storms^21^.

## Solar variability and the ionosphere

Geomagnetic storms are the results of variations in the solar currents, plasmas and the Earth’s magnetosphere which is dominated by the magnetic field. These variations are simply induced to the Earth’s surrounding by solar wind or by plasma pockets from the Sun, travelling in the solar system with their frozen in magnetic fields (i.e. CMEs or totally “Ejecta”s)^22–25^. In fact, the most extreme geomagnetic storms are associated with Coronal Mass Ejection (CME) events^26^ in most cases accompanying the solar flares.

Considering their peak fluxes, solar flares are classified as X, M and C classes ( $<10^{-4}$, $\sim 10^{-5}-10^{-4}$ and $>10^{-4}$ watts per square meter for C, M and X classes respectively). Sudden increased radiation during a solar flare, causes extra ionization of the neutral components on the day-side of the Earth’s atmosphere^27^. Whilst soft X-ray and far UV fluxes enhance ionization in the E-region, hard X-ray component is responsible for enhanced ionization in the D-region. Electrons with approximate peak energies of a few keV cause ionization in lower E region and solar proton events with energies more than 100 MeV cause ionization much deeper into the atmosphere, namely into the D-region^28–30^.

The time interval for Solar flare effects on the ionosphere, maybe divided to three main parts: (A) $0 \sim 1$ hour; increased photo ionization in the day-side, the flare energetic particles arrive shortly after the flare photons, (B) 1 hour $\sim$ 4 days; Arrival of energetic particles accelerated in fast interplanetary shocks (ICMEs), and (C) 1 day $\sim$ 4+days, the effect of interplanetary electric field on the ionospheric height on day and night-sides^31^.

The solar cycle 24, was started with low solar activity. During this “deep minimum”, the relationship between solar EUV flux and F10.7 index was deviated from its behavior in the past solar minimum^32^. Furthermore, the International Reference Ionosphere (IRI) model overestimated TEC values for this period^33–36^, thus developing semi-empirical models matters a lot.

The study of ionospheric response to solar flares, first was introduced by Afraimovich, et al.,^37–40^. In their studies, Afraimovich et al. used TEC values directly and analyzed the observed fluctuations considering variation amplitude and background fluctuations. Other researchers also studied the signature of Solar flares on TEC values^41,42^ and monitored TEC variation during geomagnetic storms^43^. These studies are essentially based on analyzing observed TEC values without trying to remove long term regular effects. In some cases the ionospheric response to solar flares was studied using more than one station^41^. Choi et al.^43^ showed ionospheric TEC variation over a region to study the response to storm periods. Instead we offer a method to study the signature of solar events even for one station. Using a semi-empirical model we produce expected hourly/daily mean values of TEC for one station during different phases of a solar cycle. Because of the applied method, these mean values represent the regular behavior of TEC. Thus, it is possible to use the results to observe the effect of irregular events such as solar flares and coronal mass ejections.

## Justification and outline of our semi-empirical model

Due to the Earth’s magnetic field, three latitudinal regions are recognized in the ionosphere: low-latitude or equatorial, mid-latitude and high-latitude regions. Usually, the low-latitude region contains the highest values of TEC whilst the mid-latitude region is considered as the least variable region of the ionosphere and it contains the most predictable variations of TEC^9^. It is shown that even in deep solar minimum a strong correlation with the solar indexes still exist^13^.

When developing a semi-empirical model, it is essential to remove the disturbed periods of geomagnetic storms^44,45^. The disturbance degree is directly related to the strength of the disturbing phenomena.

The strength of a geomagnetic storm is usually measured using geomagnetic indexes. Amongst them Kp, Dst (disturbance–storm time) ($\sim 20-30^{\circ }$ latitudes), SYM-H and ASY-H ($\sim 40-50^{\circ }$ latitudes) indexes^46–48^ provide good information about the storm condition. Generally, after the storm sudden commencement, three phases are recognized during a storm^49^: The initial (with an increase in Dst by 20 to 50 nT; for tens of minutes); main (with a Dst decreasing to less than −50 nT; 2–8 hours) and recovery phases (Dst changes from its minimum value to its quiet time value; 8 hours and above)^44^.

SYM and ASY (both -D and -H) indexes are acquired from observations of magnetic fields at low and mid-latitudes (WDC, Kyoto) and describe the development of a magnetic storm. Compression of the dayside magnetosphere in the initial phase of a storm induces positive Dst values (as well as positive SYM-H values) whilst magnetic reconnection and ring current formation induce strongly negative values during the main phase^50^. SYM-H index is considered to be an analogues of Dst in many studies^51–53^. On the other hand, it is shown that for Dst variations greater than 400 nT, these two values may differ^54^. In more detailed studies, it is recommended that SYM-H index can be used as a high-resolution Dst index^55,56^, of course with different scales for the definition of moderate storms^56^.

We have considered moderate, intense and super-storms (moderate: − 50 nT $> Dst_{ min}>$ − 100 nT ; intense: − 100 nT $> Dst_{ min}>$ − 250 nT ; super-storm: $Dst_{ min}<$ − 250 nT; During quiet times: − 20 nT $< \hbox {Dst}<$ + 20 nT) to be the most probable reason of disturbed time periods and consequently designed a suitable filter to detect and remove them.

## Database

The GPS-TEC data^57,58^ used in this research were detected by GNSS receiver Tehran (lat: 35.70N, lon: 51.33E) for a period of 11 years from low to high (2008–2013) and high to low (2014–2018) solar activity. The temporal resolution of the data is 30 seconds and received online from IONOLAB (to receive data for a time period, one can use scripting with IONOLAB-TEC/STEC Software), which provides TEC data with a resolution of 30 seconds from Receiver Independent Exchange Format (RINEX) files.

Production of ionization is mainly controlled by the solar EUV radiation. Due to unavailability of a suitable database of solar EUV radiation, solar radio flux (i.e. F10.7) is considered as a substitute index of solar activity which is reflected in our model. The daily F10.7 data were collected from OMNIWEB. Other parameters concerning IMF, solar wind and plasma parameters and activity indexes were acquired from OMNIWEB: High Resolution OMNI.

Processing the above data, the hourly/monthly mean values of the solar index (F10.7) and the ionospheric parameter TEC were prepared which allow us to study the variability of TEC with solar events.

Solar events in the above time interval were selected from Watanabe et al (2012)^59^ XRT flare catalogue and compared with the information from SOHO LASCO CME catalogue. To study the solar radio bursts RADIO SOLAR TELESCOPE NETWORK 1 Sec Solar Radio Data (SRD) files from RSTN were used.

SYM and ASY (both -D and -H) indexes are acquired from observations of magnetic fields at low and mid-latitudes (WDC, Kyoto).

## SYM-H versus Dst

It is not easy to find a unique description for the storm’s degree based on SYM-H values, specially for the common definition of a moderate storm based on Dst values. In a closer look Dst and SYM-H do not behave like each other as it is previously mentioned^55^. In some cases a moderate storm condition starts with a SYM-H index lower or higher than its Dst value (it can fluctuate around 20 nT). Thus, first we decided to re-scale SYM-H based on Dst for solar cycle 24.

A first and a simple choice was to use the same limits calculated for the period of 1985 through 2009^56^ or 1981 through 2002^55^. For instance^56^:

1 $$\begin{aligned} \textsc {sym-h}=0.95*Dst+4.5\;nT, \end{aligned}$$

gives a SYM-H index of − 43 nT for starting moderate storms (i.e. Dst of $\sim -50$ nT). A sample of “storm time intervals” consisting of the time intervals of 1000 storms (of the moderate and above degrees, picked by their Dst values) was used as the test sample for solar cycle 24. As our first step, the entire period of 2009–2018 in the search of a proper lower limit of SYM-H (i.e. for marking moderate storms and above) was studied.

**Figure 1:**
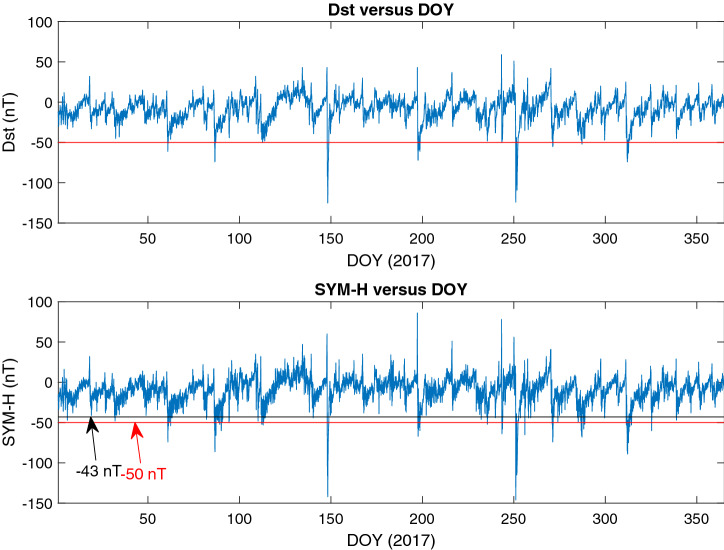
Dst and SYM-H behavior during the year 2017.

For instance, a comparison between Dst and SYM-H indexes is shown in Fig. 1, where it is seen that in the case of moderate storms, considering − 43 nT as the lower limit of SYM-H leads to the detection of more storms, so the starting value is important. Using − 46 nT gives better result for our data set in this period.

**Figure 2:**
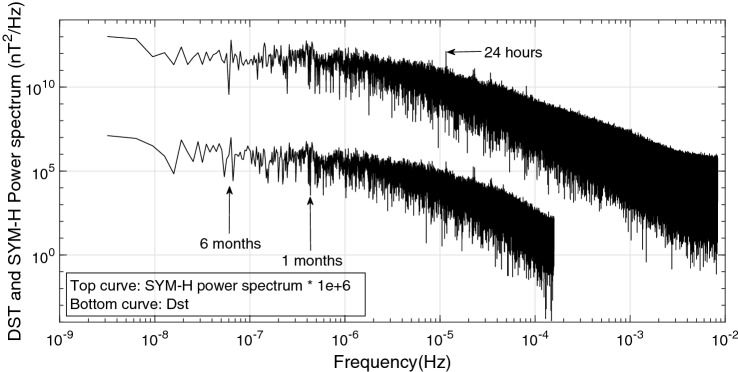
Dst and SYM-H power spectrum during 2009–2018.

Considering the behavior of power spectrums of Dst and SYM-H (Fig.  2), some differences are seen (compared with the period of 1981–2002^55^). Our power spectrums seems more noisy (Fig.  2) and as a result the peaks are not sharp as previously reported for the time interval of 1981–2002.

**Figure 3:**
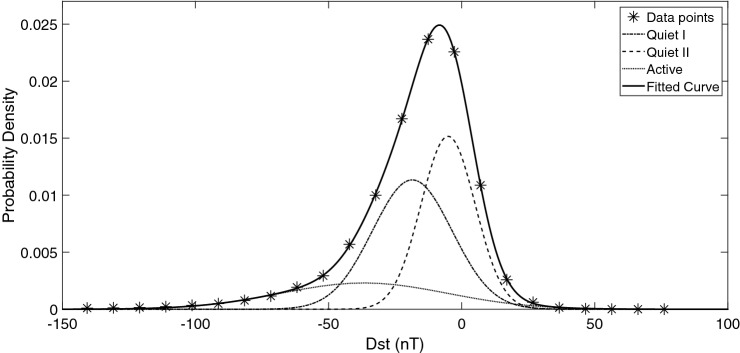
Probability Distribution Functions of Dst during 1981–2018.

**Figure 4:**
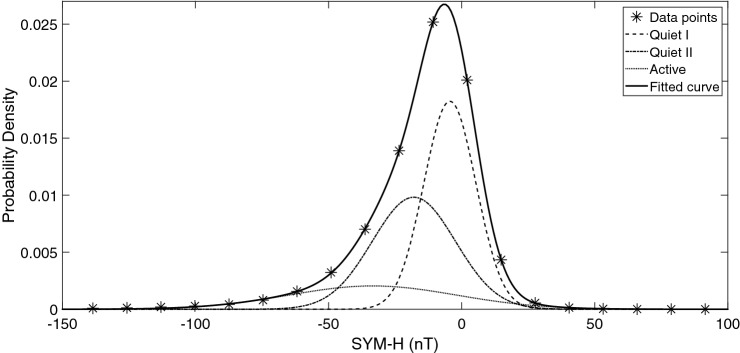
Probability Distribution Functions of SYM-H during 1981–2018.

Plotting the probability distribution functions of Dst and SYM-H also provides some information about the behavior of these values over the time interval of 1981–2018 (Figs.  3, 4).

For example it is seen that the sum of 3 gaussian distributions provides a good fit to both Dst and SYM-H distributions:

2 $$\begin{aligned} P(x) = A_1 \cdot e^{\left( x-b_1 \right) ^2 / c_1^2} + A_2 \cdot e^{\left( x-b_2 \right) ^2 / c_2^2} + A_3 \cdot e^{\left( x-b_3 \right) ^2 / c_3^2} \end{aligned}$$

Both Dst (Fig. 3) and SYM-H (Fig. 4) show 3 populations in their probability distributions, with (integrated) probability density values of: $\sim$ 0.43 ($\mu$, − 18.56 nT and $\sigma$, 14.98 nT), 0.39 ($\mu$, − 5.10 nT, and $\sigma$, 10.18 nT), 0.18 ($\mu$, − 36.72 nT, and $\sigma$, 31.32 nT) and 0.45 ($\mu$, − 4.42 nT, and $\sigma$, 9.77 nT) , 0.39($\mu$, − 17.96 nT, and $\sigma$, 15.63 nT), 0.16 ($\mu$, − 33.45 nT, and $\sigma$, 31.49 nT) respectively. In comparison the gaussian distribution with higher population (− 18.56 nT) occurs at higher SYM-H values (− 4.42 nT) and the lowest population (around $\sim$ − 30 nT) has a difference of 3 nT in the peak position for Dst and Sym-H whilst showing higher values of $\sigma$ (i.e. 31.32 and 31.49 nT) compared with two other distributions. In comparison, only less than $\sim$ − 10 percent of the events were recorded in the domain of − 50 nT and lower.

Compared with the method used by Wansliss and Showalter^55^, we see three spectrums. One Active and two Quiets (QI and QII). QI and QII cross the Active spectra at − 46nT and − 28nT respectively.

Through correlation studies for the time period of 1960–2001, the behavior of geomagnetic indices are shown to be correlated the best with Interplanetary Magnetic Fields (IMF)^60^ embedded within the solar wind. Solar magnetic field originates in convention layer and extends into the corona and the solar wind. Fast solar wind originates from coronal holes whilst slow solar wind originates at the edge of coronal holes^61^. Solar wind carries the strongest fields at solar maximum which are due to interplanetary coronal mass ejections and at this period the Earth experiences a broad range of solar wind velocities^62,63^. Around solar minimum, the coronal holes are located at the poles. When the magnetic quadrupole moment dominates over the dipole moment, a number of coronal holes appear at mid-latitudes, this is a typical behaviour in a solar cycle during solar maximum. At solar cycle 24 a deeper decrease of dipole component occurred in solar minimum^63^. During the deep solar minimum between cycles 23 and 24, the evolution of coronal holes and its connection to solar wind speed is discussed in details^64^ and a secondary peak in solar wind speed distribution is seen for 2007–2008. During solar cycle 24, solar wind speed is shown to have the highest correlation with geomagnetic indices, Ap and Dst, with zero time delays^65^. Jackson et al.^66^, used Current Sheet-Source Surface (CSSS) model^67^ to determine Geocentric Solar Magnetospheric (GSM) $B_{z}$ field. They found that the daily variations of $B_{z}$ are also correlated with geomagnetic Kp and Dst index variations over 11-year period of National Solar Observatory Global Oscillation Network Group (data. GONG). Thus it seems that the existence of 2 quiet spectrum in Figs.  3 and  4 is not accidental and may reflect the different situation of the solar cycle 24.

**Figure 5:**
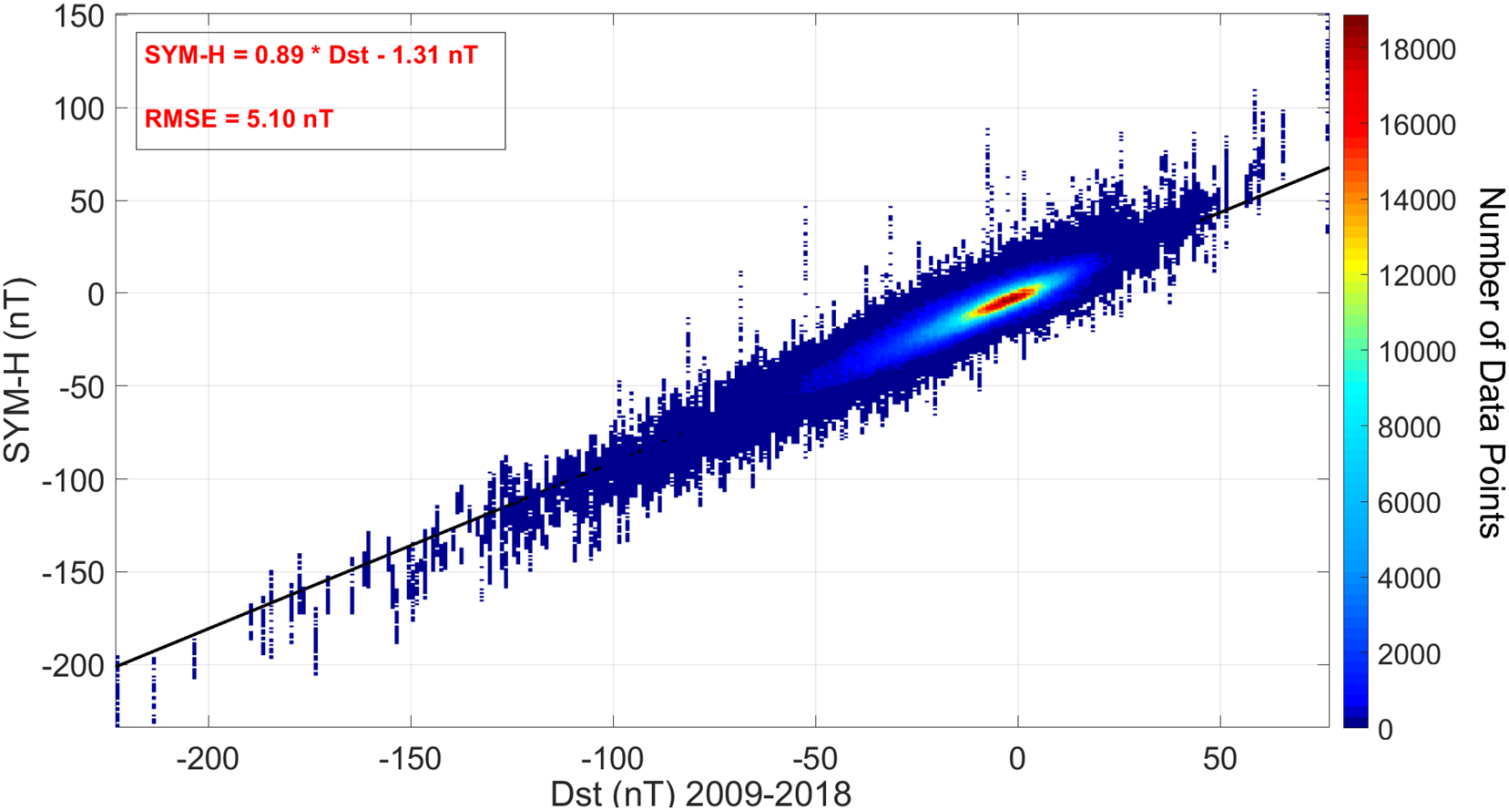
The Distribution of storm times SYM-H versus Dst during 2009–2018. The best fit line is shown in black and equation in red.

More numerical studies help to decide about the filters for executing disturbed time intervals. A linear fit to the scatter plot of SYM-H versus Dst (Fig.  5) gives the linear relationship:

3 $$\begin{aligned} \textsc {sym-h}=0.89*Dst-1.31\;nT, \end{aligned}$$

Inserting the limit of − 50 nT for Dst gives the limit of SYM-H $\sim$ − 45.81 nT, comparable with the method applied by Katus et. al.^56^ (i.e. formula  1).

## Applied filter

Instead of considering Dst variations^68^, our storm finding procedure is based on the variation of SYM-H (formula  3) whilst ASY-H is used to find the storm onset times.

**Figure 6:**
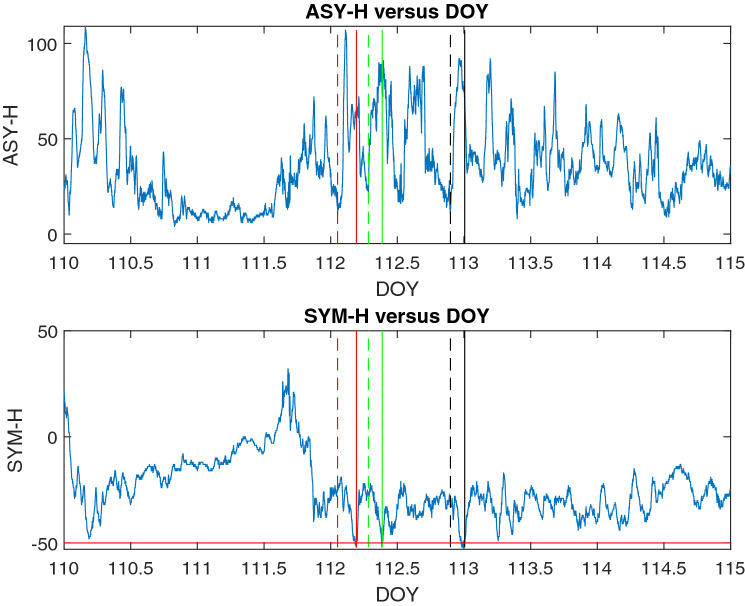
ASY-H and SYM-H behavior during geomagnetic storm of 22 April 2017 (DOY 112). The filter discussed in the section "Applied Filter"   were applied to mark disturbed time intervals. Solid lines (black, green and red) show the first occurrence of SYM-H value bellow − 46 nt. Dashed lines (black, green, red) show the start of related sub-storms considering the variation of ASY-H.

Different steps of our filtering algorithm are listed as below:

- Generally, SYM-H values below − 46 nT were considered as the start of a possible storm.
- A second filter is considered to detect storm onset times more precisely using ASY-H and SYM-H values. Observing a sharp positive peak in ASY-H values shows the sub-storm onsets^69^ prior to / or after occurrence of a moderate geomagnetic storm^70,71^. So in this method the onset times of the storms were highlighted observing the behavior of ASY and SYM (especially H) indexes.
- For the storm time periods if there is only one record with the value of “at or below” − 46 nT in the selected period, the time period was not removed.
- The recovery time also is considered (forwarded in time from the selected starting point) using ASY-H, up to the deepest local minimum after the starting point (if it does not result in less than 2 hours).
- The specified time periods were recorded and then excluded from raw data of TEC before calculating the desired mean values.

We examined our procedure for a test sample of storm time intervals during 1/1/2017–1/1/2018. The coincidence was 91.2 $\%$ using only steps 1 and 2, 97 $\%$ when adding step 3.

The above method is applied upon the whole time interval of cycle 24, backward in time. It is obvious that in comparison with the method applied by Badeke et al.^68^, we remove less time periods (they have removed 36 hours for each considered storm).

As an example of how this procedure works, the time interval of a geomagnetic storm (22 April 2017) (SGAS Number 112 Issued at 0254z on 22 Apr 2017) is shown in Fig.  6.

To study this storm a time period of 5 days is drawn (2 days before and 2 days after the reported date). The data was acquired from WDC, Kyoto.

Applying this method, in the first step 58 points were recognized with a SYM-H value below − 46 nT.

The first recognized occurrence of a SYM-H value below − 46 nT is on DOY 113, 23 April 2017, 00:08:00. Prior to this time, considering the variation of ASY-H, the start of a sub-storm is recognized on 22 April 2017, 21:33:00. Thus, the onset time for this storm was 22 April 2017, 21:33:00 and the time period from 4/22/2017 21:33:00 to 4/23/2017 00:08:00 was excluded.

Getting Back on time, the next recognized occurrence of a SYM-H below − 46 nT is on 4/22/2017 09:17:00. Same as above, the time period from 4/22/2017 06:48:00 to 4/22/2017 09:17:00 was excluded.

As the last time interval, a SYM-H below − 46 nT was recognized on 4/22/2017 04:38:00. The onset time was 4/22/2017 01:15:00 and the time period from 4/22/2017 01:15:00 to 4/22/2017 04:38:00 was excluded.

Thus, for geomagnetic storm of 22 April 2017 (above example), a sum of 08:27:00 hours was excluded from TEC raw data.

## The model

The first step to interpret the observed values of TEC, is considering a linear relation with solar F10.7 index:

4 $$\begin{aligned} \mathbf{TEC} = \varvec{\mathcal {M}} \times \varvec{F}_{10.7} + \varvec{\mathcal {B}} \end{aligned}$$

$\varvec{\mathcal {M}}$ is the dependance rate of F10.7 and $\varvec{\mathcal {B}}$ is the hypothetical value of TEC for F10.7 = 0 SFU^72^.

Semiannual, monthly and diurnal effects induce powerful variations in power spectrums of Dst and Sym-H (Fig.  2). It is expected that such regular time variations can possibly be observed in the mean values of TEC. As a result, we have calculated proper mean values for different hours (1:24) of each month of the solar cycle 24 (1:132).

**Figure 7:**
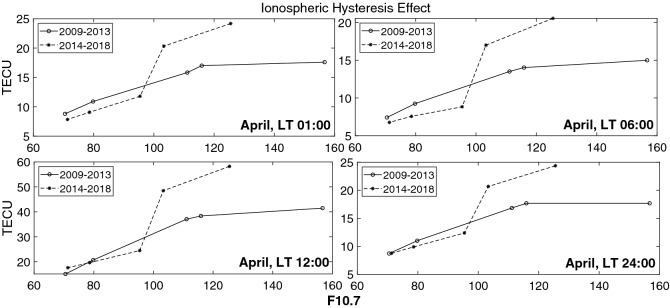
Ionospheric Hysteresis Effect for the time period of 2009–2018.

**Figure 8:**
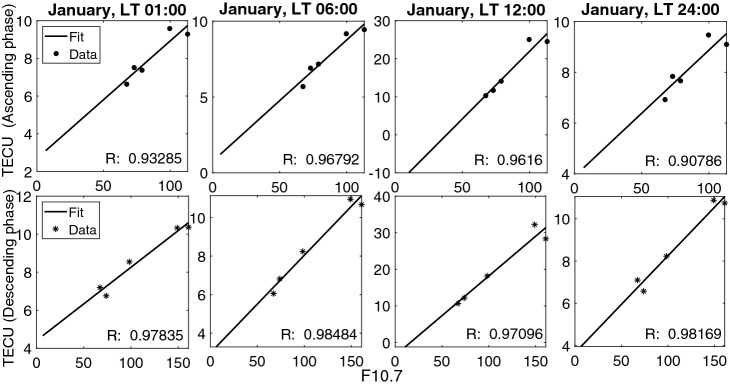
Linear regression fittings and correlation coefficients for the monthly mean values of TEC versus F10.7.

Diagram of Monthly mean values of TEC versus F10.7 solar flux, shows different behavior in ascending and descending phases of the solar cycle (i.e. the ionospheric hysteresis effect), Fig.  7. Following the present work, we intend to compare the situation of the twenty-fourth and twenty-third solar cycles. Thus, instead of dividing the twenty-fourth cycle into four periods (i.e. 2009–2011 ascending, 2011–2014 high, 2015–2016 descending and 2017–2019 low solar activity), we decided to look at a more general situation. Considering Fig.  7, the moderate linear behavior of solar cycle 24 is seen in descending phase, whilst a good correlation is obvious in ascending phase (see Fig.  8).

Thus, the whole data period has been classified in the ascending (2009–2013) and descending (2014–2018) phases of the solar cycle and the monthly mean values of TEC are calculated.

The coefficients of Eq. 4 were calculated for every hour of a day in a given month by linear regression. For each part (i.e. descending and ascending phases), a 12*24 (12 months of the year $\times$ 24 hours of the day) matrix for $\varvec{\mathcal {M}}$ and $\varvec{\mathcal {B}}$ is generated. For example in Fig. 8, the linear regression and correlation coefficients (R) between monthly mean values of TEC and F10.7 during January at different local times were shown for ascending and descending phases.

**Figure 9:**
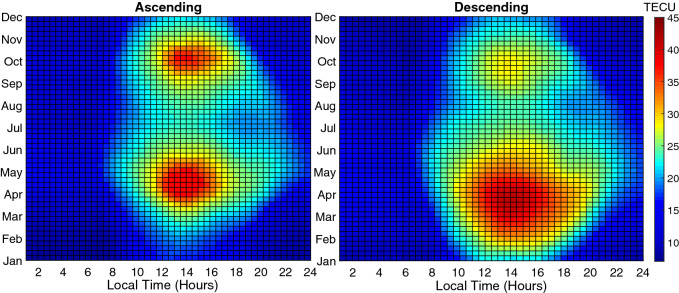
Contour diagram of the Monthly mean GPS-TEC normalized at F10.7 = 100 SFU for various months at different local times during the ascending phase (left panel) and the descending phase (right panel) of the solar cycle 24. The colour scale on the right indicates the different levels of TEC.

Contour plots of TEC, normalized at F10.7 = 100 SFU, for various months at different local times are shown in Fig. 9, for ascending and descending phases. Note that lower values of TEC are expected in comparison with similar plots for equatorial stations.

**Figure 10:**
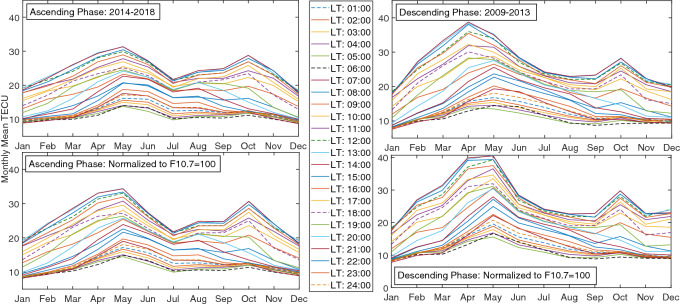
Diagrams of Monthly mean TECU in ascending and descending phases (top-left and right) and Monthly mean TECU normalized at F10.7 = 100 SFU during ascending and the descending phases (bottom-left and right).

**Figure 11:**
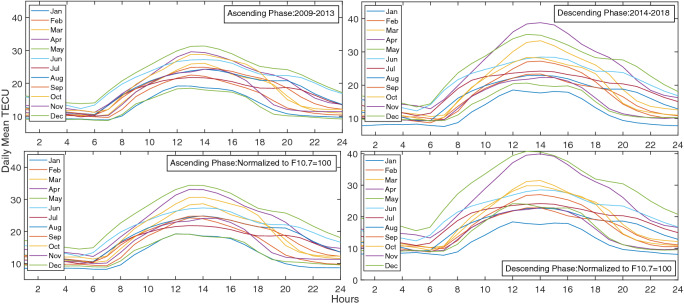
Diagrams of daily mean TECU in ascending and descending phases (top-left and right) and Monthly mean TECU normalized at F10.7 = 100 SFU during ascending and the descending phases (bottom-left and right).

The seasonal variation displays a semiannual variation with higher values around equinoxes and lower values around solstices. For instance, a comparison is made in Fig. 10 for ascending and descending phases which shows an asymmetric double peak in seasonal variation. In Fig. 11 daily mean TECU in ascending and descending phases were compared. An important feature of this semiannual variation is the local time dependence of the asymmetrical peak amplitudes. Seasonal anomaly is explained in terms of changes in solar zenith angle and thermospheric composition, especially the ratio $\frac{[O]}{[N_2]}$^73,74^.

Considering seasonal and diurnal normalized values of TEC, a sinusoidal behavior is observed during ascending and descending phases. This is the reason Fourier analysis is used in the following.

Fourier analysis was used to investigate the relation of regression coefficient matrixes, $\varvec{\mathcal {M}}$ and $\varvec{\mathcal {B}}$ with month and hour numbers. To achieve positive values for the elements of $\varvec{\mathcal {B}}$ matrix, restricted linear regression method is used.

Considering $\varvec{\mathcal {M}}$ and $\varvec{\mathcal {B}}$ as two images, all the image processing techniques is possible to be applied for further investigation.

Simply, we work with an image for which the discrete values of m and t are spatial coordinates. In the following 2D discrete Fourier analysis, is used.

If $\varvec{\mathcal {M}}(m,t)$ represents the values of matrix elements resulted by Eq. 4, then a 2D discrete Fourier transform of $\varvec{\mathcal {M}}(m,t)$ is shown by:

5 $$\begin{aligned} \varvec{\mathcal {F}}_{\varvec{\mathcal {M}}}(u,v) = \sum _{m=0}^{M-1} \sum _{t=0}^{T-1} \varvec{\mathcal {M}}(m,t) e^{-i2\pi \left( \frac{um}{M}\right) } e^{-i2\pi \left( \frac{vt}{T} \right) } \end{aligned}$$

for which *M* and *T* are equal to the dimension of our matrixes, 12 and 24 respectively. The inverse 2D discrete Fourier transform which reproduces the original matrix now is:

6 $$\begin{aligned} \varvec{\mathcal {M}}(m,t) =\frac{1}{MT} \sum _{u=0}^{M-1} \sum _{v=0}^{T-1} \varvec{\mathcal {F}}_{\varvec{\mathcal {M}}}(u,v) e^{+i2\pi \left( \frac{um}{M}\right) } e^{+i2\pi \left( \frac{vt}{T} \right) } \end{aligned}$$

Applying the same method for $\varvec{\mathcal {B}}$ and considering suitable filters to remove noises from high frequency signals, our linearized model ( 4) is extended as below:

7 $$\begin{aligned} \begin{aligned} \mathbf{TEC} (m,t)&= \frac{1}{MT} \sum _{u=0}^{M-1} \sum _{v=0}^{T-1} \varvec{\mathcal {F}}_{\varvec{\mathcal {M}}}(u,v) e^{+i2\pi \left( \frac{um}{M}\right) } e^{+i2\pi \left( \frac{vt}{T} \right) } \times \mathbf{F} _{10.7} \\&\quad + \frac{1}{MT} \sum _{u=0}^{M-1} \sum _{v=0}^{T-1} \varvec{\mathcal {F}}_{\varvec{\mathcal {B}}}(u,v) e^{+i2\pi \left( \frac{um}{M}\right) } e^{+i2\pi \left( \frac{vt}{T} \right) } \end{aligned} \end{aligned}$$

where $\varvec{\mathcal {F}}_{\varvec{\mathcal {M}}}(u,v)$ and $\varvec{\mathcal {F}}_{\varvec{\mathcal {B}}}$ represent the Fourier coefficients of 2D discrete Fourier expansion of equations like  6.

Using proper low and high-pass filters it is possible to decompose the original matrix to the components of desired frequency bands. This method is suitable to study the main frequencies in our linearized model. Here we have applied low-pass filters to remove high frequency noises and the main frequencies were used to reconstruct TEC values. Thus the model is re-written using new values of $\varvec{\mathcal {M}}(m,t)$ and $\varvec{\mathcal {B}}(m,t)$ (respectively, $\varvec{\mathcal {G}}_1(m,t)$ and $\varvec{\mathcal {G}}_2(m,t)$)).

8 $$\begin{aligned} \mathbf{TEC} = \varvec{\mathcal {G}}_1(m,t) \times \mathbf{F} _{10.7} + \varvec{\mathcal {G}}_2(m,t). \end{aligned}$$

**Figure 12:**
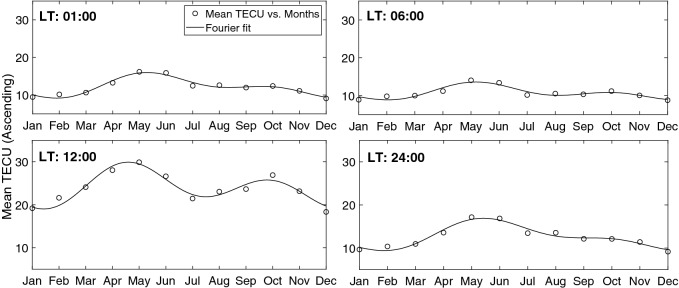
Seasonal variation of monthly mean normalized TEC values at different local times during the ascending phase of the solar cycle 24 (2009–2013). The continuous thick curve presents the resulted fourier series.

**Figure 13:**
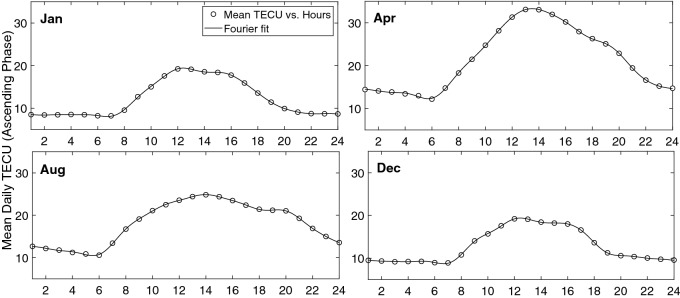
Seasonal variation of daily mean normalized TEC values at different months during the ascending phase of the solar cycle 24 (2009–2013). The continuous thick curve presents the resulted fourier series before using filters.

**Figure 14:**
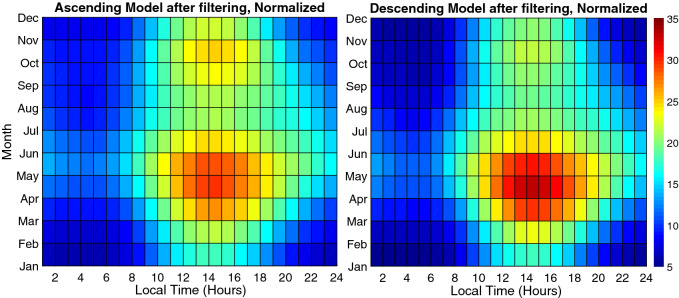
Final Result of Ionospheric model - Normalized to F10.7 = 100 SFU.

**Figure 15:**
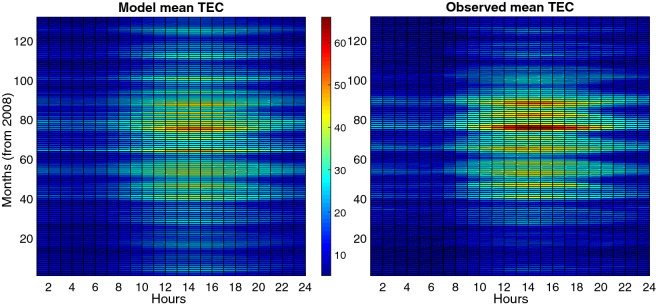
Final Result of Ionospheric model for the entire cycle. Left panel represents the result of our semi-empirical model, whilst right panel represents mean values of observed TECs. The model response is best for higher solar activities.

Applying this model, it is possible to compare resulting values with the observed values. Thus, for solar cycle 24 we have marked a set of time intervals for which, a positive residual exist.

In our method the accuracy of the time interval was one hour, but it is possible to increase the accuracy to one minute by considering proper mean values of experimental GPS-TEC (i.e. Fig.  9).

A few examples of our resulted TEC values at four different local times, 1, 6, 12, and 24 during ascending phase of solar cycle 24 are shown in Figs. 12 and  13.

Figure 13 shows diurnal variation of monthly mean values of TEC for different months of the ascending phase. It is clear that TEC gradually increases with sunrise, reaches a peak at around 11:00–14:00 LT and later declines to reach the minimum value after midnight.

The final results of the model are presented in Figs.  14 and  15 . Figure  14 shows the normalized mean values of TEC for ascending and descending phases, whilst Fig.  15 demonstrates the observed mean values for Tehran station in comparison with the model mean values.

Now it is possible to study the effects of CME and solar flares on TEC values. As an example, the impact of X-class solar flares is investigated in the following section.

## The impact of X-class solar flares of cycle 24

We apply our method to the full data-set without removing the disturbed time intervals (as in Sect. 6), resulted values are represented by TEC(E), in Table  2. Thus TEC(E), will possibly contain both regular and irregular effects 1. We also presented values estimated by our full criteria (Sect. 6), represented by TEC(MM) in Table  2. Finally, the resulted values are compared against TEC values of a standard plasmasphere-ionosphere model (International Reference Ionosphere, IRI), represented by TEC(I) in Table  2.

**Figure 16:**
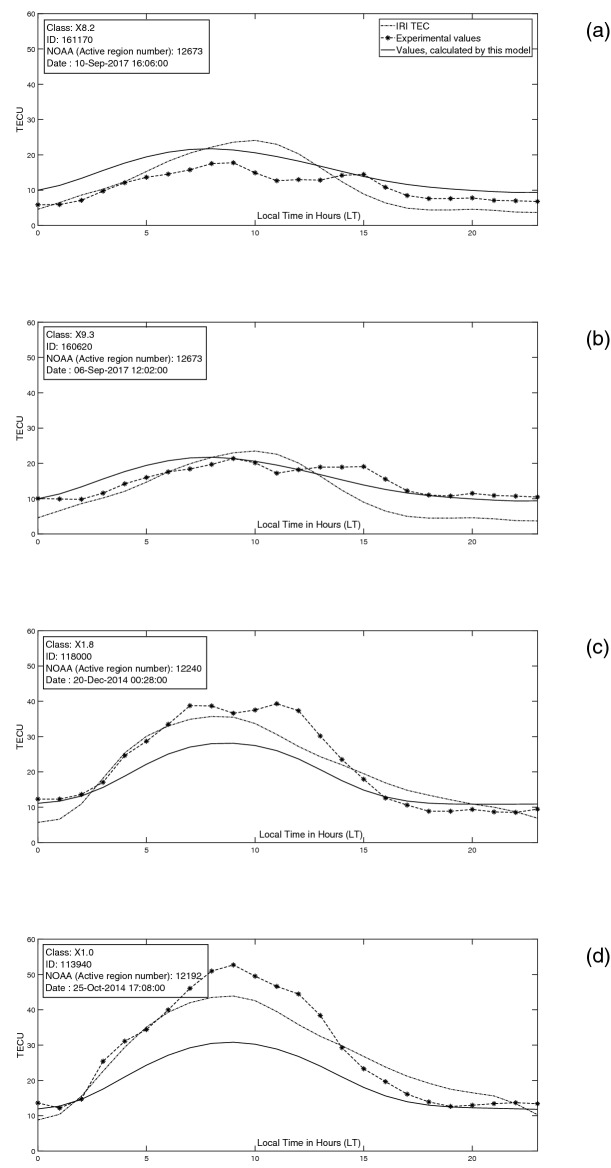
Daily variation of TECU for few different flares.

Our results for four flare events, are shown in Fig. 16a–d. In comparison to IRI model, our model results in smoother curves for TEC.

**Table 1:** List of studied solar event.

ID	CLASS	YY	MM	DD	Daily F10.7 flux
031070	X2.2	11	02	15	110.0
033440	X1.5	11	03	09	141.0
041780	X6.9	11	08	09	100.2
043810	X2.1	11	09	06	113.2
048150	X1.9	11	11	03	157.8
053820	X1.7	12	01	27	137.4
055540	X1.1	12	03	05	129.5
055910	X5.4	12	03	07	133.7
055920	X1.3	12	03	07	133.7
064660	X1.1	12	07	06	163.0
065220	X1.4	12	07	12	170.9
071530	X1.8	12	10	23	140.1
082890	X1.7	13	05	13	153.5
082980	X2.8	13	05	13	153.5
083010	X3.2	13	05	14	151.1
083050	X1.2	13	05	15	148.8
092540	X1.1	13	11	10	151.1
102140	X1.0	14	03	29	142.3
104230	X1.3	14	04	25	126.2
111580	X1.6	14	09	10	177.0
113350	X1.1	14	10	19	171.7
113710	X1.6	14	10	22	214.2
113870	X3.1	14	10	24	215.4
113940	X1.0	14	10	25	216.8
114000	X2.0	14	10	26	214.0
114190	X2.0	14	10	27	185.4
115000	X1.6	14	11	07	142.9
118000	X1.8	14	12	20	196.7
122740	X2.1	15	03	11	129.9
126290	X2.7	15	05	05	130.0
160610	X2.2	17	09	06	134.9
160620	X9.3	17	09	06	134.9
160720	X1.3	17	09	07	130.4
161170	X8.2	17	09	10	101.6

**Table 2:** Our results compared with the results of IRI model (2016) for solar event of tabel 1. TEC(E), TEC(MM) and TEC(I) represent experimental, calculated by this model and calculated by IRI model values of TEC.

TEC(I)	TEC(E)	TEC(MM)	TEC(E)-TEC(I)	TEC(E)-TEC(MM)	Daily F10.7 flux	CLASS	ID	Date-Time
319.400	338.274	384.060	18.874	− 45.786	110.000	X2.2	031070	15-Feb-2011 01:45:00
387.700	0.000	576.814	− 387.700	− 576.814	141.000	X1.5	033440	09-Mar-2011
465.000	448.301	454.660	− 16.699	− 6.359	100.200	X6.9	041780	09-Aug-2011 08:05:00
500.000	0.000	543.307	− 500.000	− 543.307	113.200	X2.1	043810	06-Sep-2011
524.700	569.648	500.229	44.948	69.419	157.800	X1.9	048150	03-Nov-2011 20:27:00
458.800	411.510	372.706	− 47.290	38.804	137.400	X1.7	053820	27-Jan-2012 18:37:00
608.400	494.013	560.822	− 114.387	− 66.809	129.500	X1.1	055540	05-Mar-2012 04:05:00
630.800	595.659	560.822	− 35.141	34.837	133.700	X5.4	055910	07-Mar-2012 00:24:00
630.800	595.659	560.822	− 35.141	34.837	133.700	X1.3	055920	07-Mar-2012 01:14:00
490.800	670.332	626.538	179.532	43.794	163.000	X1.1	064660	06-Jul-2012 23:08:00
481.500	537.416	626.538	55.916	− 89.122	170.900	X1.4	065220	12-Jul-2012 16:49:00
479.500	469.093	626.538	− 10.407	− 157.445	140.100	X1.8	071530	23-Oct-2012 03:17:00
640.800	891.365	615.657	250.565	275.708	153.500	X1.7	082890	13-May-2013 02:17:00
640.800	891.365	615.657	250.565	275.708	153.500	X2.8	082980	13-May-2013 16:01:00
640.400	833.747	615.657	193.347	218.090	151.100	X3.2	083010	14-May-2013 01:11:00
639.700	774.173	615.657	134.473	158.516	148.800	X1.2	083050	15-May-2013 01:40:00
633.700	501.158	459.236	− 132.542	41.922	151.100	X1.1	092540	10-Nov-2013 05:14:00
768.500	1022.878	771.702	254.378	251.176	142.300	X1.0	102140	29-Mar-2014 17:48:00
788.900	934.813	739.271	145.913	195.542	126.200	X1.3	104230	25-Apr-2014 00:27:00
620.500	659.693	633.176	39.193	26.517	177.000	X1.6	111580	10-Sep-2014 17:33:00
655.500	0.000	471.949	− 655.500	− 471.949	171.700	X1.1	113350	19-Oct-2014
661.700	590.041	471.949	− 71.659	118.092	214.200	X1.6	113710	22-Oct-2014 14:06:00
636.700	617.843	471.949	− 18.857	145.894	215.400	X3.1	113870	24-Oct-2014 21:15:00
635.000	668.495	471.949	33.495	196.546	216.800	X1.0	113940	25-Oct-2014 17:08:00
632.900	175.708	471.949	− 457.192	− 296.241	214.000	X2.0	114000	26-Oct-2014 10:56:00
631.000	592.129	471.949	− 38.871	120.180	185.400	X2.0	114190	27-Oct-2014 14:23:00
611.000	621.306	606.158	10.306	15.148	142.900	X1.6	115000	07-Nov-2014 17:26:00
487.000	518.782	421.102	31.782	97.680	196.700	X1.8	118000	20-Dec-2014 00:28:00
656.200	659.615	650.272	3.415	9.343	129.900	X2.1	122740	11-Mar-2015 16:22:00
655.800	0.000	571.799	− 655.800	− 571.799	130.000	X2.7	126290	05-May-2015
279.900	354.448	360.955	74.548	− 6.507	134.900	X2.2	160610	06-Sep-2017 09:10:00
279.900	354.448	360.955	74.548	− 6.507	134.900	X9.3	160620	06-Sep-2017 12:02:00
280.800	425.313	360.955	144.513	64.357	130.400	X1.3	160720	07-Sep-2017 14:36:00
283.800	265.420	360.955	− 18.380	− 95.535	101.600	X8.2	161170	10-Sep-2017 16:06:00

For completeness of the research, we studied the time intervals of class X solar flares in solar cycle 24^59^ (XRT flare catalogue). Some of these flares are companying with CMEs. During the studied dates the possibility of solar radio bursts from (RSTN) also are considered in which 1 second records of eight discrete solar radio flux measurement were presented. A set of 34 flares of class X were considered for the present work (Table  1). For Tehran station, no TEC values existed for 4 flares out of 34 (Table  1), and TEC values existed only partially for 2 flares out of 34.

In a total view, in 19 events (out of 34) the expected mean values of TEC from the presented model are somewhat lower than IRI 2016.

Figure  16a, demonstrate the TEC variation of 10-Sep-2017. An X8.2 flare with ID: 161170 (Watanabe et al (2012)^59^ XRT flare catalogue) is seen at 16:06:00 UT with a CME accompanying (SOHO/LASCO Halo CME Catalogue). Though it was one of the most powerful events in solar cycle 24, its effect on TEC values was not considerable for Tehran station, just a second peak reached to our calculated values. Figure  16b, demonstrate the TEC variation of 06-Sep-2017. An X9.3 flare with ID: 160620 (Watanabe et al (2012)^59^ XRT flare catalogue) is seen at 12:02:00 UT with a CME accompanying (SOHO/LASCO Halo CME Catalogue). A second peak in TEC, is seen for $\sim$ 14 to 15 pm in Fig.  16b, and experimental TEC values are well above our calculated values. Solar event of 10-Sep-2017, is well modeled^75^. Though the CME eruption was catalogued as Halo, with a Central Position Angle (CPA) of 360 degrees (SOHO/LASCO CME Catalogue), it does not produced an Earth directed Interplanetary CME (ICME). During this event, three CMEs propagated and merged into a complex ICME with main direction towards Mars^75,76^. So despite of an X8.3 flare, solar events were not geoeffective and excited no Forbush Decrease (FD) or powerful Geomagnetic Storms (GMSs). In comparison, as we see in Fig.  16b, solar flare of 6-Sep-2017, was accompanying with a Halo CME affected with two prior CMEs from the same active region (SOHO/LASCO CME Catalogue). The interaction of these ICMEs together and with high speed stream from coronal hole 823 and corotating interaction region and heliospheric current sheet^76^ resulting strong IMF and solar wind.

Third example (Fig.  16c) represents the effect of a class X1.8 solar flare, on 20-Dec-2014. For flare ID: 118000 (Watanabe et al (2012)^59^ XRT flare catalogue), no halo CME event is recorded by SOHO/LASCO Halo CME Catalogue. The flare occurrence is recorded on 00:28 UT and TEC values at Tehran station is disturbed dramatically around noon. Due to (SOHO/LASCO CME Catalogue) 6 CMEs are recorded from $\sim$ 1 am to 21 pm. A partial halo CME occurred at 01:25 am with a CPA of 216 degrees. This event is not followed more, but possibility of a G1 minor geomagnetic storm was reported (Space Weather Prediction Center).

**Figure 17:**
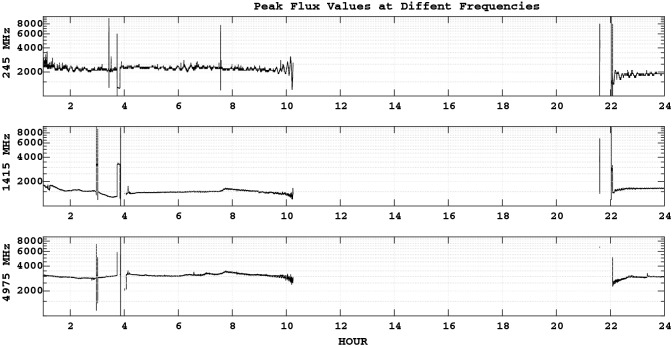
Peak flux values at 3 different frequencies: 245, 1415 and 4975 MHz (25-Oct-2014).

Our last example, Fig.  16d, shows TEC values in the occurrence date of an X1.0 solar flare (25-Oct-2014). For flare ID: 113940 (Watanabe et al (2012)^59^ XRT flare catalogue), no halo CME event is recorded by SOHO/LASCO Halo CME Catalogue. TEC values are disturbed again around noon, where it is clear that this later event is not connected by flare ID: 113940 at 17 pm (Watanabe et al (2012)^59^ XRT flare catalogue). A partial halo CME erupted at 4 am (SOHO/LASCO CME Catalogue) Geomagnetic field was forecasted to be quiet to unsettled (Space Weather Prediction Center). The solar burst data (RSTN) is used to observe solar burst in 8 frequencies of 245, 410, 610, 1415, 2695, 4975, 8800 and 15400 MHz. The peak values is shown in Fig. 17.

For the time interval of 10 am to 22 pm no data exist in all 8 frequencies, but there are few bursts which seems to be responsible for TEC variations of Fig.  16d.

Due to this study, it is seen that a combination of solar events are responsible for TEC variations. But the effect of Solar flares and bursts with radio emissions higher than daily F10.7 values, were detected more clearly at the mid-latitude station of Tehran (the situation might be quite different, e.g., at high latitude stations).

Our resulted values shows that the flares with the most power (i.e. class X) have induced a variation of more than 20 percent in TEC. In some cases for the flares with accompanying CMEs, the variation maybe extended to a few hours. In our study we found no correlation between the local time of the flares occurrence and the magnitude of induced values.

## The results and conclusions

- The probability density of Dst and SYM-H (Figs. 3 and  4) for the time interval of 1981–2018 were formulated as the sum of 3 gaussian distributions:
  9 $$\begin{aligned} P(x) = A_1 \cdot e^{\left( x-b_1 \right) ^2 / c_1^2} + A_2 \cdot e^{\left( x-b_2 \right) ^2 / c_2^2} + A_3 \cdot e^{\left( x-b_3 \right) ^2 / c_3^2} \end{aligned}$$
  shows 3 populations based of the magnitude of ionospheric disturbances.
- For the solar cycle 24, SYM-H was re-scaled based on Dst as (Fig.  5):
  10 $$\begin{aligned} \textsc {sym-h}=0.89*Dst-1.31\;nT, \end{aligned}$$
- A method to recognize the time intervals of geomagnetic storms based on SYM-H and ASY-H variations is developed and applied in the section Applied Filter.
- The final results of the model are presented in Figs.  14 and  15 .
- The same calculation method is used to present estimated values of IRI 2016 model as a reference.
- Solar flares of class X in solar cycle 24 were studied for completeness. Figure  16a,b and Tables  1 and  2.
- In the absence of any halo CME, Earth directed ICMEs and streamers from the Sun, solar radio bursts of 25-Dec-2014 (Fig.  17) are probable source of TEC variations (Fig.  16d).
- Through this method the effect of Solar flares and bursts with radio emissions higher than daily F10.7 values, were better detected.
- Our resulted values shows that the flares with the most power (i.e. class X) have induced a variation of more than 20 percent in TEC.
